# Factors Associated with Length of Hospitalization in Patients with Diabetes and Mild COVID-19: Experiences from a Tertiary University Center in Serbia

**DOI:** 10.3390/medicina60050788

**Published:** 2024-05-09

**Authors:** Vojislav M. Ciric, Natasa Krsto Rancic, Milica M. Pesic, Danijela B. Radojkovic, Nikola Milenkovic

**Affiliations:** 1 Faculty of Medicine Nis, University of Nis, 18000 Nis, Serbia; ciricv@yahoo.com (V.M.C.); pesic@medfak.ni.ac.rs (M.M.P.); danijela.radojkovic@medfak.ni.ac.rs (D.B.R.); 2 Universital Clinical Center Nis, Clinic for Endocrinology, Diabetes and Metabolic Diseases, 18000 Nis, Serbia; milenkovic@dr.com; 3 Institute for Public Health Nis, Center for Diseases Control and Prevention, 18000 Nis, Serbia

**Keywords:** type 2 diabetes mellitus, COVID-19, length of hospitalization

## Abstract

*Background and Objectives*: During the COVID-19 pandemic, there was an increased number of hospitalized COVID-19-positive patients suffering from type 2 diabetes mellitus (T2DM). The objective of this research study was to explore factors associated with the length of hospitalization of patients with T2DM and the mild form of COVID-19. *Material and Methods*: This retrospective cohort study involved all patients who tested positive for COVID-19 and those who were treated in the dedicated COVID-19 department of the University Clinical Center (UCC) in Nis between 10 September 2021 and 31 December 2021. Upon admission, patients underwent blood tests for biochemical analysis, including blood count, kidney and liver function parameters (C-reactive protein (CRP), creatinine kinase, and D-dimer), and glycemia and HbA1c assessments. Additionally, all patients underwent lung radiography. Univariate and multivariate regression analyses were employed to assess the impact of specific factors on the length of hospitalization among patients with T2DM. *Results*: Out of a total of 549 treated COVID-19-positive patients, 124 (21.0%) had T2DM, while 470 (79.0%) did not have diabetes. Among patients with T2DM, men were significantly younger than women (60.6 ± 16.8 vs. 64.2 ± 15.3, *p* < 0.01). The average hospitalization length of patients with diabetes was 20.2 ± 9.6 (5 to 54 days), and it was significantly longer than for patients without diabetes, at 15.0 ± 3.4, which ranged from 3 days to 39 (*t*-test ≈ 5.86, *p* < 0.05). According to the results of the univariate regression analysis, each year of age is associated with an increase in the length of hospital stay of 0.06 days (95% CI: 0.024 to 0.128, *p* = 0.004). Patients who received oxygen therapy were treated for 2.8 days longer than those who did not receive oxygen treatment (95% CI: 0.687 to 4988, *p* = 0.010), and each one-unit increase in CRP level was associated with a 0.02-day reduction in the length of hospitalization (95% CI: 0.004 to 0.029, *p* = 0.008). Based on the results of the multivariate regression analysis, each year of age is associated with an increase in the length of hospitalization by 0.07 days (95% CI: 0.022 to 0.110, *p* = 0.003). Patients who received oxygen therapy were treated for 3.2 days longer than those who did not receive oxygen therapy (95% CI: 0.653 to 5726, *p* = 0.014), and each unit increase in CRP level was associated with a 0.02-day reduction in the length of hospitalization (95% CI: 0.005 to 0.028, *p* = 0.004). *Conclusions*: Based on the presented results, COVID-19-positive patients with diabetes had, on average, longer hospitalizations than COVID-19 patients without diabetes. The hospital treatment of patients with T2DM and a milder form of COVID-19 was associated with older age, the use of oxygen therapy, and elevated CRP values. Patients who received oxygen therapy were treated approximately 3 days longer than those who did not receive this therapy.

## 1. Introduction

COVID-19 is a global health crisis that has placed a significant burden on healthcare systems worldwide [1]. Patients with diabetes mellitus (DM) are a particularly vulnerable population facing an increased risk of severe outcomes from COVID-19 infection, including prolonged hospitalization periods. DM, a chronic condition affecting the body’s metabolic balance, can compromise the immune response and hinder the recovery process from infections [2].

In December 2019, cases of severe pneumonia with unknown etiology appeared in the human population in China [2]. The confirmation on 7 January 2020 was related to the identification of SARS-CoV-2 as the new causative agent of coronavirus disease 2019 (COVID-19). COVID-19 rapidly spread from China to the entire world; the World Health Organization (WHO) confirmed the human–human transmission of the SARS-CoV-2 virus on 23 January 2020 and declared a pandemic on 11 March 2020 [3,4]. Based on data from the WHO, there are cumulatively over 762 million confirmed cases of COVID-19 and more than 6.9 million deaths worldwide [5]. During the same period, according to the European Centre for Disease Prevention and Control in the EU/EEA territory, over 275 million confirmed cases of infection have been reported [6].

According to one meta-analysis, an overall pooled prevalence of 31% of DM in hospitalized COVID-19 patients [7] and those with type 2 diabetes mellitus (T2DM) is estimated. DM has been identified as the major comorbidity of hospitalized patients with COVID-19 [8,9,10]. The prevalence of DM in COVID-19 patients ranges from 5% to 36% [11,12]. People with diabetes are a particularly vulnerable group of patients regarding infections [13], and during COVID-19, it was demonstrated that DM is a pro-inflammatory syndrome characterized by an inadequate cytokine response, with significantly higher concentrations of interleukin-6 (IL-6), ferritin, and C-reactive proteins in the blood compared with COVID-19-positive patients without diabetes [14,15,16]. It has been shown that uncontrolled glycemia in patients with diabetes increases their vulnerability to inadequate immune responses relative to infection, further exacerbating the disease and leading to the development of acute respiratory distress syndrome (ARDS) and shock. The determined and increased secretion of proinflammatory cytokines in patients with diabetes may change the nature of the immune response related to SARS-CoV-2 infection into inflammation, increasing the probability of a severe course of COVID-19 and resulting in a cytokine storm and tissue and respiratory damage [15,16]. Good glycemic control may be important for maintaining the balance of the immune system [17].

Several studies have found associations between diabetes and greater odds of adverse clinical outcomes in COVID-19, including a longer length of hospitalization, higher mortality rates and intensive care unit (ICU) admissions, and the use of mechanical ventilation [18,19,20]. Moreover, based on the results of one retrospective analysis of 211,003 medical records, COVID-19-positive patients with T2DM had twice the risk of hospitalization [19]. Gottlieb et al. [20] and Martos-Benirez et al. [21] reported similar results. In a large cross-sectional study in Mexico, COVID-19-positive patients with DM were 38.4% more likely to be hospitalized; moreover, the estimated probability was higher if associated diseases were present. Halalau et al. [22] conducted a multicenter study to show a significant increase in the hospitalization of COVID-19-positive patients suffering not only from DM but also from pre-diabetes.

Based on the findings from a study from the United States of America (USA) with almost a million participants, 20.5% of COVID-19 hospitalizations were attributable to DM. It is estimated that a 10% reduction in DM prevalence could reduce hospital admissions for COVID-19 infection by 2.7% [23]. Based on the results of a large study from the USA, DM was the second most frequent comorbidity, corresponding to 15% of COVID-19-infected individuals, while the prevalence of DM in the same region was 9.7% [24]. T2DM was also reported as an independent risk factor for ICU admission after adjustments for age, sex, comorbidities, and insurance status [25]. Moreover, in a large cohort of hospitalized COVID-19 participants, patients with T2DM had higher rates of intensive care unit (ICU) admission and intubation and longer hospital stays compared with patients without diabetes [26].

DM has not been proven to be an independent factor in the hospitalization of COVID-19-positive patients [20]. The heterogeneity in the design of the aforementioned studies and differences in local policies for COVID-19 spread prevention may be responsible for the lack of consensus. In addition to associated diseases, factors contributing to the hospitalization of patients with diabetes include older age, male sex, and delayed medical care and treatment [18,19,20,21,22].

The first confirmed case of COVID-19 in Serbia was registered on 6 March 2020, and the epidemic of greater epidemiological significance was declared on 19 March 2020. By the end of 2022, a total of 2,446,253 cases and 17,519 deaths were confirmed [27]. According to the Center for Disease Control (CDC) [28], the dominant strain of the virus in 2021 was the Delta variant. The Delta variant was initially identified in India and quickly spread to other countries. It was a significant factor in the increase in COVID-19 cases in many regions, as it was considered to be highly contagious. Omicron was first identified in Botswana and South Africa in late November 2021, and cases quickly began to surface and multiply in other countries. By December 2021, the original Omicron strain (BA.1) was no longer circulating, and Omicron subvariants are now driving most SARS-CoV-2 infections.

The main objective of this research study was to explore factors associated with the length of hospitalization in patients with T2DM and the mild form of COVID-19 in one tertiary university center in Serbia.

## 2. Material and Methods

### 2.1. Study Design

A retrospective cohort study in a single tertiary center was performed, and it included all COVID-19-positive patients treated in the COVID-19 department of the University Clinical Center (UCC) in Nis from 10 September 2021 to 31 December 2021. The total number of COVID-19 patients with a milder form of COVID-19 was 549 (according to the criteria in Table 1), and they were not hospitalized in the intensive care unit (ICU) of the hospital. After obligatory testing for SARS-CoV-2 polymerase chain reaction (PCR), the values of glycemia were checked using the oral glucose tolerance test (OGTT). Based on the results of this test and other medical documentation of the patients, all patients were divided into two groups: T2DM COVID-19-positive and only COVID-19-positive patients.

The study was carried out following the Declaration of Helsinki and the decisions of the University Clinical Center’s (UCC’s) Ethics Committee (decision number 28827/2; dated 20 October 2020).

### 2.2. Data Sources and Patient Admission Procedure

Epidemiological, descriptive, clinical, and laboratory data were taken from the electronic records of hospitalized patients with COVID-19. Upon admission to the COVID-19 department, patients’ medical histories were taken, and all patient characteristics, clinical symptoms of COVID-19, and the method of taking samples for COVID-19 testing were recorded. Moreover, upon admission, patients with COVID-19 had their blood taken for biochemical analysis, and the determination of blood counts and kidney and liver function parameters was carried out: C-reactive protein, creatinine kinase, D-dimer, glycemia, and HbA1c. The oral glucose tolerance test was carried out in some patients with specific symptoms: dry mouth, polydipsia, and high blood glucose. Medical documentation was obtained. All patients underwent lung radiography. The patients were administered specific COVID-19 therapy and oxygen therapy, and the vaccination status against COVID-19 was checked.

### 2.3. Statistical Analysis

All collected data were entered into the electronic database, and the software package SPPS version 22.0 was used for data processing. Univariate and multivariate regression analyses were used to determine the influence of certain factors on the length of hospitalization with respect to patients with T2DM.

## 3. Results

Of the total number of treated COVID-19-positive patients, there were 124 (21.0%) with T2DM and 470 (79.0%) without diabetes. Men with T2DM were significantly younger than women (60.6 ± 16.8 vs. 64.2 ± 15.3, *p* < 0.01). Patients with T2DM had no significant differences in gender and age with respect to patients without diabetes (*p* > 0.01). There were significantly more patients with T2DM who were vaccinated against COVID-19, at 70 patients (56.5%), than patients without diabetes, at 142 patients (30.1%). The average hospitalization length in patients with diabetes was 20.2 ± 9.6 (5 to 54 days), and it was significantly longer than in patients without diabetes, at 15.0 ± 3.4, which ranged from 3 days to 39. The serum levels of CRP were much higher in patients with diabetes, ranging from 2.2 to 325.0 (and creatinine ranged from 45.0 to 771.4) than in those without diabetes (CRP ranged from 1.0 to 224.0; creatinine ranged from 42.0 to 663.0). The diabetes group had significantly higher average serum levels of CRP than the group without diabetes (*p* = 0.008). Patients with diabetes had a significantly higher rate of pre-existing comorbidities (65.0% vs. 48%) than patients without diabetes.

Table 2 shows the univariate analysis results of the most significant factors impacting the length of hospitalization of patients with T2DM and a mild form of COVID-19.

According to the results of the univariate regression analysis, each year of age is associated with an increase in the length of hospitalization by 0.07 days (95% CI: 0.024 to 0.128 days, *p* = 0.004). Patients who received oxygen therapy were treated 2.8 days longer than those who did not receive oxygen treatment (95% CI: 0.687 to 4.988, *p* = 0.010), and each one-unit increase in CRP level was associated with a 0.02-day reduction in the length of hospitalization (95% CI: 0.4 to 0.029, *p* = 0.008).

Based on the results of the multivariate regression analysis, each year of age is associated with an increase in the length of hospitalization by 0.07 days (95% CI: 0.022 to 0.110, *p* = 0.003). Patients who received oxygen therapy were treated 3/2 days longer than those who did not (95% CI: = 0.653 to 5.726, *p* = 0.014), and each unit increase in CRP level was associated with a 0.02-day reduction in the length of hospitalization (95% CI: 0.005 to 0.028, *p* = 0.004).

Only 5% of the variability in hospitalization duration was determined using the characteristics examined in this research study (coefficient of determination: R^2^ = 0.050).

## 4. Discussion

Based on the results of our study, COVID-19-positive patients with diabetes had, on average, longer hospitalization durations than COVID-19 patients without diabetes. The length of hospitalization of T2DM patients with mild COVID-19 was associated with older age, the use of oxygen therapy, and elevated CRP values. Women with T2DM were significantly older than men.

Based on the findings of Al-Salameh et al., COVID-19 patients with diabetes had a higher risk of death, higher ICU admission rates, and prolonged hospital stays [30]. Our results correspond to these results and the findings from COVID-19 hospitals in Serbia. According to the findings of the COVID-19 hospital in Batajnica, Serbia, better metabolic control upon admission was associated with shorter hospital stay durations and lower mortality rates in COVID-19-positive patients with diabetes, which implies the importance of prior glycemic control among patients with T2DM and CKD [31]. In a COVID-19 hospital in Zvezdara, out of 3664 patients, 960 (26.2%) had DM, which accounted for more than a quarter of the patients; moreover, in over half of them, DM persisted for longer than 10 years [32].

There are also different findings available.

According to Kisic et al., among the total number of examined COVID-19-positive patients, 68.6% exhibited a comorbid condition, whereas 57.5% had pre-existing cardiovascular disease (CVD). Statistical analyses confirmed that comorbidities did not significantly influence the duration of hospitalization [33]. According to the findings of Alabai et al. (2024), SatO_2_, glycemia at hospital admission, and HbA1c had the highest sensitivity and specificity in predicting the prognosis of T2DM patients with SARS-CoV-2 infection. Glycemic control is essential in the prognosis of patients with DM and COVID-19 infection [34]. Our results indicated that age was one of the factors influencing longer hospitalization durations among COVID-19-positive patients with diabetes.

In our study, women were significantly older than men and had longer hospitalization durations. Although the pathophysiological mechanisms are still not understood, they may be explained by the dysfunction of the immune system with aging. Age-specific rates per 100,000 inhabitants by gender during 2022 in the Republic of Serbia, as a result of COVID-19, were higher in all age categories among females, with the exception of those in the age categories of 0–14 and 60 and above, where a higher rate was observed among males. The highest age-specific rates of COVID-19 incidence in the general population in Serbia were observed in the age group of 60 and above (13,556.91/100,000 inhabitants), while the lowest age-specific rate was recorded in the age group of 0–14 years, at 6525.68 per 100,000 inhabitants [35].

In a single-center descriptive study conducted over a period of 2 years (April 2020 to April 2022) in Turkey among 3050 COVID-19 patients treated in the ICU, there were findings of a potential association between age and sex, and biomarkers such as D-dimer, CRP, liver enzymes, ferritin, lactate dehydrogenase were associated with the severity of COVID-19 and prolonged hospitalization of patients [36].

During COVID-19, there were significantly fewer hospitalized children and young people with type 1 diabetes mellitus (T1DM), which indirectly suggests that they were at lower risk of severe clinical presentation and that hospitalization was not necessary [7,11,12]. An increased risk of COVID-19 in children and adults with T1DM has not been identified [34], and there is also no documented evidence of increased mortality among these patients during the COVID-19 pandemic. According to previous studies, older patients with diabetes are at a higher risk for severe COVID-19 and could eventually require ICU admission; their condition may even result in death [12,21,22,35,36].

In a study conducted in China, it was determined that COVID-19-positive patients were mostly men (60%), but more often, women exhibited elevated body temperatures and prolonged hospitalization. There were more women in the elderly population; thus, they were hospitalized more often, and the observed factor was older age with a high body temperature [37].

According to our results, one reason for prolonged hospitalization was elevated CRP levels among patients with diabetes. Similar data can be found in the literature. According to the findings of Liu et al., increased CRP levels and lymphopenia are independent risk factors for COVID-19 severity, while lymphopenia is also a risk factor for prolonged hospital stay [18].

In a study on COVID-19-positive patients with an asymptomatic clinical picture and those with mild symptoms, which was conducted by Wu et al., it was determined that patients with diabetes had elevated body temperatures upon admission, and this was the main reason for prolonged hospitalization [37]. The results of a retrospective study conducted in Vietnam reported that age, the place of permanent residence, and sources of infection were significantly associated with the prolonged hospitalization of patients with T2DM and COVID-19 during the second wave of COVID-19 [38].

Based on our findings, patients with diabetes who received oxygen therapy were treated approximately 3 days longer than those who did not receive this form of therapy. According to the results of a study conducted in Korea, DM was not an independent predictor of the severity of the clinical picture of the disease, which was defined as the need for oxygen therapy and/or artificial ventilation [19]. DM was a predictor of ICU admission, the occurrence of COVID-19 complications, and prolonged hospitalization duration [39,40,41,42]. A study in western Sydney reported that patients with diabetes had a 6% increase in mortality, an 8% increase in ICU requirement, and a 6.6-day increase in the length of stay (*p* < 0.01) [43]. Based on the finding of a study by Luviano-Garcı’a et al. (2024), the variables associated with a greater risk of requiring intubation after high-flow therapy were age (HR = 1.018, 95% CI: 1.003–1.034, *p* = 0.022) and body mass index (BMI) (HR = 1.071, 95% CI: 1.024–1.120, *p* = 0.003) [44].

The control measures during COVID-19 and the illness itself have impacted the deterioration of the health of patients with T2DM in several ways: reduced mobility due to restrictions on outings in some countries due to lockdowns, the irregular supply of dietary food and medication for diabetes and associated conditions, and disruptions in regular medical check-ups and glycemic control. Many patients with COVID-19 symptoms delayed seeking medical attention and testing due to the fear of isolation, hospitalization, and separation from their families. There has been an increase in anxiety and depression among this group of patients and the entire population.

Limitations of the study: Limitations commonly observed in studies on the length of hospitalization due to COVID-19 include small sample sizes, insufficient data, and temporal constraints.

The limitations of our small sample include insufficient representativeness, an increased risk of chance effects, and difficulty in identifying rare events. The other limitations of the study are that laboratory biochemical tests were not performed for all patients, and it was not possible to evaluate all important parameters. There is a lack of data on COVID-19 vaccination statuses for all hospitalized individuals. The interpretation of the results can also be affected by the sample size. In order to avoid mistakes, we took a sample of patients with diabetes who were treated in the largest healthcare facility in the city of Nis and the entire territory of southeastern and southern Serbia.

Power of the study: This is the first study to determine factors that contribute to hospitalization and the length of hospitalization of patients with diabetes and a mild clinical form of COVID-19.

## 5. Conclusions

Based on the presented results, the length of hospital treatment of patients with diabetes and a milder form of COVID-19 was associated with older age, the use of oxygen therapy, and elevated CRP values. Patients who received oxygen therapy were treated approximately 3 days longer than those who did not receive therapy. Understanding these factors is crucial for adjusting medical care and improving outcomes for these patients during the pandemic.

## Figures and Tables

**Table 1:** Criteria of the mild form of COVID-19 based on the World Health Organization: COVID-19 disease severity [29].

Mild COVID-19 Illness
Symptomatic patients satisfying the case definition for COVID-19 without evidence of hypoxia or pneumonia.
Common symptoms include fever, cough, fatigue, anorexia, dyspnea, and myalgia. Other non-specific symptoms include sore throat, nasal congestion, headache, diarrhea, nausea/vomiting, and loss of smell/taste. Additional neurological manifestations reported include dizziness, agitation, weakness, seizures, or findings suggestive of stroke. Children may not report fever or cough as frequently as adults.
Older people and immunosuppressed people may present atypical symptoms (e.g., fatigue, reduced alertness, reduced mobility, diarrhea, loss of appetite, delirium, and absence of fever).
Symptoms due to physiological adaptations of pregnancy, adverse pregnancy events (e.g., dyspnea, fever, gastrointestinal symptoms, and fatigue), or other diseases (e.g., malaria) may overlap with COVID-19 symptoms.

**Table 2:** Correlations between the length of hospitalization and other investigated characteristics; the results of univariate and multivariate regression analyses.

Characteristics	R	95% CI Limits		*p*
		Lower	Upper	
Univariate regression analysis				
Men	−1.004	−2.700	0.692	0.245
Age (years)	0.076	0.024	0.128	0.004
T2DM	1.189	−1.073	3.451	0.302
Vaccine	0.803	−0.916	2.522	0.359
Oxygen therapy	2.837	0.687	4.988	0.010
COVID-19-specific Th	0.180	−3.930	4.289	0.932
HbA1c	−1.864	−4.261	0.533	0.122
CRP	−0.017	−0.029	−0.004	0.008
Creatinine	−0.009	−0.019	0.002	0.100
DM duration	−0.244	−0.885	0.396	0.422
Multivariate regression analysis				
Men	−0.175	−1.572	1.222	0.805
Age (years)	0.066	0.022	0.110	0.003
T2DM	−0.952	−3.592	1.687	0.479
Vaccine	0.605	−0.920	2.130	0.436
Oxygen therapy	3.189	0.653	5.726	0.014
COVID-19-specific Th	−1.347	−5.078	2.383	0.478
HbA1c	−1.496	−3.011	0.020	0.053
CRP	−0.016	−0.028	−0.005	0.004
Creatinine	−0.008	−0.017	0.001	0.083
T2DM duration	−0.192	−0.759	0.375	0.507

## Data Availability

The dataset underlying this study is available upon reasonable request from the first author or the corresponding author.

## References

[1] World Health Organization (WHO) (2023). Weekly Epidemiological Update on COVID-19. https://www.who.int/publications/m/item/weekly-epidemiological-update-on-covid-19-23-august-202.

[2] Sharma P., Behl T., Sharma N., Singh S., Grewal A.S., Albarrati A., Albratty M., Meraya A.M., Bungau S. (2022). COVID-19 and diabetes: Association intensify risk factors for morbidity and mortality. Biomed. Pharmacother..

[3] (2019). Report of Clustering Pneumonia of Unknown Etiology in Wuhan City. Wuhan Municipal Health Commission. http://wjw.wuhan.gov.cn/front/web/showDetail/2019123108989.

[4] World Health Organization (2020). Transmission of SARS-CoV-2: Implications for Infection Prevention Precautions, Scientific Brief. https://www.who.int/publications/i/item/modes-of-transmission-of-virus-causing-covid-19-implications-for-ipc-precaution-recommendations.

[5] World Health Organization Coronavirus (COVID-19) Dashboard. https://covid19.who.int/.

[6] ECDC (2023). COVID-19 Situation Update Worldwide. https://www.ecdc.europa.eu/en/covid-19/situation-updates.

[7] Bradley S.A., Banach M., Alvarado N., Ivica Smokovski I., Bhaska S.M.M. (2022). Prevalence and impact of diabetes in hospitalized COVID-19 patients: A systematic review and meta-analysis. J. Diabetes.

[8] Singh A.K., Gupta R., Ghosh A., Misra A. (2020). Diabetes in COVID-19: Prevalence, pathophysiology, prognosis and practical considerations. Diabetes Metab Syndr..

[9] Alguwaihes A.M., Al-Sofiani M.E., Megdad M., Albader S.S., Alsari M.H., Alelayan A., Alzahrani S.H., Sabico S., Al-Daghri N.M., Jammah A.A. (2020). Diabetes and COVID-19 among hospitalized patients in Saudi Arabia: A single-centre retrospective study. Cardiovasc. Diabetol..

[10] Guo M., Li Y., Dong H., Zhou Z., Zhang C., Tian R., Qin H., Wang Y., Shen K., Du L. (2020). Diabetes is a risk factor for the progression and prognosis of COVID-19. Diabetes Metab. Res Rev..

[11] Rees E.M., Nightingale E.S., Jafari Y., Waterlow N.R., Clifford S., Pearson C.A.B., Jombart T., Procter S.R., Knight G.M., CMMID Working Group (2020). COVID-19 length of hospital stay: A systematic review and data synthesis. BMC Med..

[12] Pijls B.G., Jolani S., Atherley A., Derckx R.T., Dijkstra J.I., Franssen G.H., Hendriks S., Richters A., Venemans-Jellema A., Zalpuri S. (2021). Demographic risk factors for COVID-19 infection, severity, ICU admission and death: A meta-analysis of 59 studies. BMJ Open.

[13] Knapp S. (2013). Diabetes and infection: Is there a link?—A mini-review. Gerontology.

[14] Petrie J.R., Guzik T.J., Touyz R.M. (2018). Diabetes, hypertension, and cardiovascular disease: Clinical insights and vascular mechanisms. Can. J. Cardiol..

[15] Geerlings S.E., Hoepelman A.I. (1999). Immune dysfunction in patients with diabetes mellitus (DM). FEMS Immunol. Med. Microbiol..

[16] Moutschenm M.P., Scheenm A.J., Lefebvrem P.J. (1992). Impaired immune responses in diabetes mellitus: Analysis of the factors and mechanisms involved. Relevance to the increased susceptibility of diabetic patients to specific infections. Diabete Metab..

[17] Geza T., Wojtowicz K., Guzik P., Góra T. (2022). Increased Risk of COVID-19 in Patients with Diabetes Mellitus—Current Challenges in Pathophysiology, Treatment and Prevention. Int. J. Environ. Res. Public Health.

[18] Liu X., Zhou H., Zhou Y., Wu X., Zhao Y., Lu Y., Tan W., Yuan M., Ding X., Zou J. (2020). Risk factors associated with disease severity and length of hospital stay in COVID-19 patients. J. Infect..

[19] You J.H., Lee S.A., Chun S.Y., Song S.O., Lee B.W., Kim D.J., Boyko E.J. (2020). Clinical Outcomes of COVID-19 Patients With Type 2 Diabetes: A Population-Based Study in Korea. Endocrinol. Metab..

[20] Hernández-Galdamez D.R., González-Block M.Á., Romo-Dueñas D.K., Lima-Morales R., Hernández-Vicente I.A., Lumbreras-Guzmán M., Mendez-Hernandez P. (2020). Increased Risk of Hospitalization and Death in Patients With COVID-19 and Pre-Existing Noncommunicable Diseases and Modifiable Risk Factors in Mexico. Arch. Med. Res..

[21] Gottlieb M., Sansom S., Frankenberger C., Ward E., Hota B. (2020). Clinical Course and Factors Associated With Hospitalization and Critical Illness Among COVID-19 Patients in Chicago, Illinois. Acad. Emerg. Med..

[22] Martos-Benitez F.D., Soler-Morejon C.D., Garcia-Del Barco D. (2021). Chronic Comorbidities and Clinical Outcomes in Patients With and Without COVID-19: A Large Population-Based Study Using National Administrative Healthcare Open Data of Mexico. Intern. Emerg. Med..

[23] Halalau A., Odish F., Imam Z., Sharrak A., Brickner E., Lee P.B., Foglesong A., Michel A., Gill I., Qu L. (2021). Epidemiology, Clinical Characteristics, and Outcomes of a Large Cohort of COVID-19 Outpatients in Michigan. Int. J. Gen. Med..

[24] O’Harem M., Liu J., Cudhea F., Micha R., Mozaffarin D. (2021). Coronavirus Disease 2019 Hospitalizations Attributable to Cardiometabolic Conditions in the United States: A Comparative Risk Assessment Analysis. J. Am. Heart Assoc..

[25] Xu G., Liu B., Sun Y., Du Y., Snetselaar L.G., Hu F.B., Bao W. (2018). Prevalence of Diagnosed Type 1 and Type 2 Diabetes Among US Adults in 2016 and 2017: Population Based Study. BMJ.

[26] Huang J.F., Wang X.B., Zheng K.I., Liu W.Y., Chen J.J., George J., Zheng M.H. (2020). Obesity hypoventilation syndrome and severe COVID-19. Metabol. Clin. Exp..

[27] Huang A.A., Huang Y.S. (2023). Hospitalized COVID-19 patients with diabetes have an increased risk for pneumonia, intensive care unit requirement, intubation, and death: A cross-sectional cohort study in Mexico in 2020. Health Sci Rep..

[28] Centers for Disease Control and Prevention (2020). Epidemiological Triad. Atlanta, Georgia: Centers for Disease Control and Prevention.

[29] World Health Organization (2021). Living Guidance for Clinical Management of COVID-19. https://apps.who.int/iris/handle/10665/338882.

[30] Al-Salameh A., Lanoix J.P., Bennis Y. (2021). Characteristics and outcomes of COVID-19 in hospitalized patients with and without diabetes. Diabetes Metab. Res Rev..

[31] Stoiljkovic M., Lalic N., Macesic M., Stanarcic Gajovic J., Mina Milovacevic M., Spurnic A., Adzic-Vukicevic T. Type 2 Diabetes and Chronic Kidney Disease: COVID-19 Outcomes. Proceedings of the First World Conference “Fighting COVID-19 Pandemic—Health Challenges”.

[32] Kisic G., Petrovic T., Peric V. Impact of Comorbidities on the Length and Course Hospitalisation in Patients with COVID-19. Proceedings of the First World Conference “Fighting COVID-19 Pandemic—Health Challenges”.

[33] Vuksanovic M., Jojic B., Marjanovic Petkovic M., Stojanovic J., Andjelic Jelic M., Beljic Zivkovic T. Glycemic Control and Vaccinal Status of Hospitalized Patients with Diabetes in Covid Hospital KBC Zvezdara. Proceedings of the First World Conference “Fighting COVID-19 Pandemic—Health Challenges”.

[34] Albai O., Braha A., Timar B., Sima A., Deaconu L., Timar R. (2024). Assessment of the Negative Factors for the Clinical Outcome in Patients with SARS-CoV-2 Infection and Type 2 Diabetes Mellitus. Diabetes Metab. Syndr. Obes..

[35] Institute of Public Health of Serbia ‘Dr. Milan Jovanović Batut’ (2023). Report on Infectious Diseases in the Republic of Serbia for the Year 2022.

[36] Genc S., Taghizadehghalehjoughi A., Naldan M.E., Gülcü O., Caglayan C., Spanakis M., Nikolouzakis T.K., Alegakis A., Docea A.O., Drocas A.I. (2024). Evaluation of various blood biomarkers associated with the outcomes of patients with COVID-19 treated in intensive care units. Exp. Ther. Med..

[37] Wu S., Xue L., Legido-Quigley H., Khan M., Wu H., Peng X., Li X., Li P. (2020). Understanding factors influencing the length of hospital stay among non-severe COVID-19 patients: A retrospective cohort study in a Fangcang shelte hospital. PLoS ONE.

[38] Do T.V., Manabe T., Vu G.V., Nong V.M., Fujikura Y., Phan D., Pham T.T., Do C.D., Doan T.T., Nguyen N.T. (2023). Clinical characteristics and mortality risk among critically ill patients with COVID-19 owing to the B.1.617.2 (Delta) variant in Vietnam: A retrospective observational study. PLoS ONE.

[39] O’Malley G., Ebekozien O., Desimone M., Pinnaro C.T., Roberts A., Polsky S., Noor N., Aleppo G., Basina M., Tansey M. (2021). COVID-19 hospitalization in adults with type 1 diabetes: Results from the T1DM Exchange Multicenter Surveillance Study. J. Clin. Endocrinol. Metab..

[40] Corrao S., Argano C., Natoli G., Nobili A., Corazza G.R., Mannucci P.M., Perticone F., RePoSi Investigators (2019). Sex Differences in the Pattern of Comorbidities, Functional Independence, and Mortality in Elderly Inpatients: Evidence from the RePoSI Register. J. Clin. Med..

[41] Richardson S., Hirsch J.S., Narasimhan M., Crawford J.M., McGinn T., Davidson K.W., Barnaby D.P., Becker L.B., Chelico J.D., Cohen S.L. (2020). Presenting Characteristics, Comorbidities, and Outcomes Among 5700 Patients Hospitalized with COVID-19 in the New York City Area. JAMA.

[42] Moon S.J., Rhee E.J., Jung J.H., Han K.D., Kim S.R., Lee W.Y., Yoon K.H. (2020). Independent Impact of Diabetes on the Severity of Coronavirus Disease 2019 in 5307 Patients in South Korea: A Nationwide Cohort Study. Diabetes Metab. J..

[43] Cheung N.W., Gilroy N., Hor A., Jose S., Kairaitis K., Nayyar V., O’Sullivan M.V., Wheatley J., Chipps D.R. (2022). Diabetes and hyperglycaemia among hospitalizeed patients with COVID-19 in Western Sydney: A retrospective cohort study. Intern. Med. J..

[44] Luviano-García J.A., Loose-Esparza A., Hernández-Ruíz Y.G., Sanz-Sánchez M.Á., Maheda-García H.J., Sosa-Medellin M.A., Garza-Silva A., Romero-Ibarguengoitia M.E. (2024). Risk factors for intubation and mortality in patients treated with high flow nasal cannula due to COVID-19 infection. Survival Analysis Study in a Northern Mexican Population. PLoS ONE.

